# Smart-Autonomous Wireless Volatile Organic Compounds Sensor Node for Indoor Air Quality Monitoring Application

**DOI:** 10.3390/ijerph19042439

**Published:** 2022-02-20

**Authors:** C. Bambang Dwi Kuncoro, Moch Bilal Zaenal Asyikin, Aurelia Amaris

**Affiliations:** 1 Department of Refrigeration, Air Conditioning and Energy Engineering, National Chin-Yi University of Technology, Taichung 41170, Taiwan; bilalmoch@gmail.com; 2 Department of Informatics Engineering, Faculty of Computer Science, Universitas Brawijaya, Malang 65145, Indonesia; aureliamariss@gmail.com

**Keywords:** IAQM, TVOCs, sensor node, low power

## Abstract

Several studies reported the significant effect of indoor air quality on human health, safety, productivity, and comfort because most humans usually conduct 80%–90% of their activity inside the building. This is generally due to the fact that indoor pollution is associated with volatile organic compounds (VOCs), pollutants with chronic health effects, both non-carcinogenic and carcinogenic, on humans. Therefore, this study focused on developing wireless VOCs sensor nodes with a low-power strategy feature to perform an autonomous operation in indoor air quality monitoring (IAQM). The sensor node mainboard consists of a microcontroller-based AVR (ATmega-4808) that supports a low power mode and low-power IAQ-Core sensor for VOCs detection. The low-power sensing algorithm developed also allowed the sensor node to consume a total power of 0.22 mAh for one cycle of operation, which includes the initial process, TVOCs value reading process, data transmitting process, and low power mode process at a time interval of 30 min. The most significant power was observed to be consumed in the data transmitting process with 0.13 mAh or 58% of total power consumption in one cycle of sensor node operation. Furthermore, the 10F capacitance of the supercapacitor was able to drive the VOCs sensor node for 139 s and it was recommended that further studies use micro energy harvesting (from an indoor environment) to extend its lifetime. The 1541-minute field experiment conducted also showed that TVOCs and CO_2_ values were successfully measured and displayed over an internet connection on the monitoring terminal dashboard. The recorded real-time TVOCs value of 175 ppb (<200 ppb) indicates good air quality.

## 1. Introduction

The 2030 Agenda for Sustainable Development pays serious attention to healthy living. This is indicated in Goal 3 (3.9), which is targeted towards reducing the number of deaths caused by pollution and contamination, as well as Goal 11 (11.1–11.3), which focuses on safety and health by gender, age, and persons with disabilities from different types of residence. Another goal (11.6–11.B) also targets air quality and solid waste in urban environments, public spaces, and cities with social and economic impacts [1]. Moreover, the development of science and technology promotes innovation in realizing healthy living with a focus on air pollution. This is considered necessary because the exploitation of natural resources, manufacturing activities, and the massive use of transportation are known to be producing unavoidable air pollution, which is very hazardous to human health and even has the ability to cause death [2,3]. Meanwhile, previous studies have shown that humans conduct 80%–90% of their activities indoors [3,4,5,6].

Indoor air pollution is usually caused by pollutants such as particulate matter (PM), volatile organic compounds (VOCs), chemical factors, and others existing in the air inside buildings. The most common form is VOCs, which are produced from hydrocarbons in materials normally used in the living environment such as aromatic compounds, liquid fuels, and paraffin [7]. VOCs are easily vaporized in the air when the vapor pressure is high and are unknowingly present in the environment all the time. It is important to note that the massive use of chemical substances needs to be monitored and avoided due to the harmful effects of the VOCs particles on human health such as throat irritation, headaches, coordination loss, fatigue, and several others [8,9]. Moreover, the concentration of VOCs indoors is higher than outdoors because those emitted inside are associated with fragrances and other consumer products [10]. This indicates there is a need to monitor these pollutants in order to increase health awareness.

Organizations such as the WHO [11], US EPA [12], and German Federal Environmental Agency [13] have issued some indoor air quality (IAQ) standards [14] to ensure continuous monitoring of indoor air pollution from all directions. Several studies focused on the optimal use of the sensor [15] in monitoring indoor air quality, while others compared its processor usage [16] and algorithm development [17,18]. In Ref. [19], particles such as carbon dioxide (CO_2_), temperature, humidity, PM_2.5_, and VOCs were considered in IAQ monitoring with their variations observed to have been monitored successfully through the system developed, which was designed for flexible implementation and to support low-cost sensors. Another study [20] also monitored CO_2_, NO_2_, HCHO, and TVOCs successfully using an ARM microcontroller-based LPC2478 equipped with automatic control of the heat exchange ventilator. The “poor” status in the monitoring system was designed to automatically trigger the heat-exchange ventilation operation and this was reported to have a better performance compared to another ventilation method, specifically the traditional natural method. Moreover, the authors of Ref. [21] developed a wireless gas sensor network for pollutant detection and explained the evolution of the prototype up to the fourth version, which is equipped with a solar panel. The sensor nodes were also powered by a Li-ion battery with a solar panel for outdoor application. The review conducted by [22] on the study of air quality monitoring between 2015 and 2020 discovered that Arduino, Raspberry, and ESP8266 modules were usually used as the main processing units. Several studies also considered using Bluetooth and the ZigBee platform instead of Wi-Fi as the communication protocol due to their power efficiency.

It was observed that most of these previous studies focus on the sensor to detect pollutants and measure air quality parameters. Meanwhile, a power strategy needs to be a concern for continuous, real-time, and stand-alone sensor node operation in air quality monitoring systems. This is essential for long-term operation or sensor node lifetime durability considering the fact that a built-in battery is normally used as its main power source. According to [23], the detection of atmospheric carbon dioxide and methane requires low power consumption with a highly sensitive sensor, and the application of the device in a different place for around nine months showed that it has a good result. It is important to note that the stand-alone status of a sensor node in an indoor air quality monitoring system sometimes makes it non-optimal to replace and substitute the battery when it runs because it is not effective, costly, and raises some environmental issues.

This present study was conducted to develop an air quality sensor node with a compact size and a new powering technique for autonomous operation in an indoor air quality monitoring (IAQM) system. The node was built based on a modular approach with the focus on the measure of the VOCs parameters in real time, after which the data are to be sent to the cloud through a transmit algorithm implemented in order to ensure low power consumption. Moreover, a dashboard placed in the monitoring terminal was designed to monitor and trace the VOCs concentration to determine the indoor air quality levels as excellent, good, moderate, poor, or unhealthy. This low-power strategy developed can be utilized in further studies in creating a VOCs sensor node system to receive input power from micro energy harvesting in the indoor environment.

## 2. Materials and Methods

### 2.1. Volatile Organic Compounds (VOCs)

VOCs are a wide group of organic chemical compounds found in several products that vaporize easily into the environment under normal conditions. They have high volatility, mobility, and resistance to degradation, thereby allowing their movement through vast distances [24]. Their concentration in the space is usually a balance between the net emissions in the area and the supplies from the ventilation such that the existence of a high concentration of TVOCs in a building possibly indicates the presence of a strong pollutant or inadequate general or local ventilation [25]. This means it is possible to determine the insufficient or poor ventilation design in a building using this factor and a higher value indicates a higher concentration level as shown in Table 1. It is important to reiterate that TVOCs usually affect the sense of well-being and comfortability inside a building. Some of their particles include methylene chloride, tetrachloroethylene, toluene, benzene, ethylene glycol, and formaldehyde.

TVOCs are measured in milligram per cubic meter (mg/m^3^) or part per billion (ppb). The conversion from mg/m^3^ to µg/m^3^ or part per million (ppm) is expressed in Equation (1) [26].

(1) $$\rho_{\mathrm{gas}\;\mathrm{mix}}\left[\mu g/m^{3}\right]=\frac{M_{gas\;mix}[g/\mathrm{mol}]}{V_{m}\times 1000\;\mathrm{ppb}}\times c_{gas\;mix}\;\left[\mathrm{ppb}\right]$$

where $\rho_{\mathrm{gas}\;\mathrm{mix}}$, $M_{gas\;mix}$, and $c_{gas\;mix}$ are the mass concentration, the average molar mass, and the particle concentration of the defined gas mix, respectively, while $V_{m}$ is the molar volume, which is 0.0244 $m^{3}$/mol at 25 °C, and the atmospheric pressure and the mean molar mass of this mixture is 110 g/mol.

### 2.2. Low-Power Strategy

A real-time VOCs monitoring requires real-time data transmission from the acquisition devices. The system developed in this study is configured to read the indoor TVOCs parameters and transmit the data to the server to be displayed in the monitoring dashboard. Meanwhile, the changes in the indoor air quality are not rapid, and this condition can be used as an opportunity to reduce power consumption through the implementation of an appropriate time strategy. This is possible because setting up the time interval to transmit data is one of the methods to ensure a low-power system considering the fact that a data logging device generally requires more power when transmitting data [27]. Moreover, high data transmitting frequency is directly proportional to power consumption. Another strategy is to use a chip that supports sleep mode when the system is not transmitting data. Some AVR chips support three sleep modes including idle, standby, and power down, which are considered suitable for the low-power system with some interfaces and features on the processor disabled during this mode to reduce the power consumption in the system.

### 2.3. Energy Storage

This study used the supercapacitor to store energy instead of a battery due to its high energy density and ability to supply the quick bursts of demanded power rapidly [28]. However, the charging process of the supercapacitor requires a regulator circuit to keep the voltage and current according to the specification considering the fact that a ripple current can reduce its lifetime [29]. The supercapacitor has been previously used in [30] to store power from a piezo electronics generator and was reported to have the ability to function in the low-power system at a power consumption unit of 67 µW. This is observed to be in line with the long-term goal of developing a VOCs sensor node system to receive input power from the energy-harvesting process in the indoor environment.

### 2.4. Design Overview

**Figure 1:**
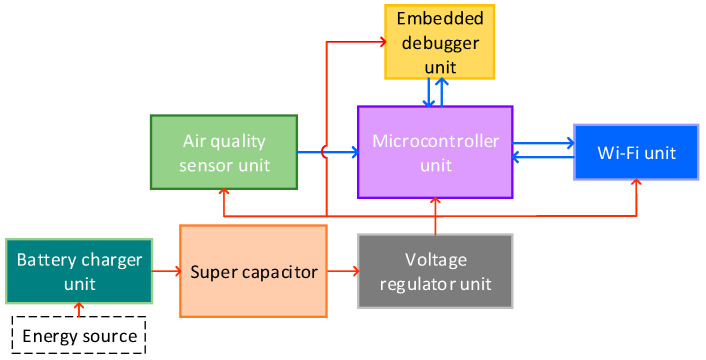
IAQ sensor node architecture.

This study developed a smart, autonomous, wireless sensor node to be applied in monitoring indoor air quality. The proposed device comprises four major units including the energy storage, sensor, processor, and wireless transceiver as shown in the functional block diagram presented in Figure 1. The entire units of the proposed device are designed to be powered by rechargeable energy storage in line with the plan for future autonomous devices coupled with an energy harvesting system. The processor used is a microcontroller-based AVR and supports low power mode. Moreover, the system is equipped with a Wi-Fi module that connects it to the internet for data transmission purposes. The power source is a supercapacitor while the external power supply is designed to be used during the charging process and released during the discharging process for the supercapacitor to function. However, the system is expected to be linked to indoor energy harvesting as a power source in the future.

#### 2.4.1. Mainboard

**Figure 2:**
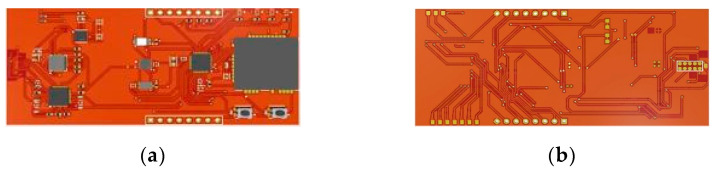
PCB design of IAQ sensor node: (**a**) top layer and (**b**) bottom layer.

The proposed device was designed to be compact, small, and work based on AVR ATmega-4808. All the system units including the power source component are integrated into the board as indicated in the 3D board design presented in Figure 2. It is also important to note that all the components used are SMD in order to achieve the low-power system design.

The system was designed to read the VOCs sensor through the I_2_C interface and send data to the cloud through Wi-Fi communication. It was also designed to work in the 3.3–5 V voltage range, thereby making the supercapacitor a suitable power source. The overall specifications of the system are presented in Table 2.

#### 2.4.2. Supercapacitor

Two parallel supercapacitors were used as the main power source with each having 5.0 F/5.5 V and the total capacity was calculated to be 10 F using Equation (2).

Capacitor in parallel connection:

C_eq_ = C_0_ + C_1_ + … + C_n_ (2)

**Figure 3:**
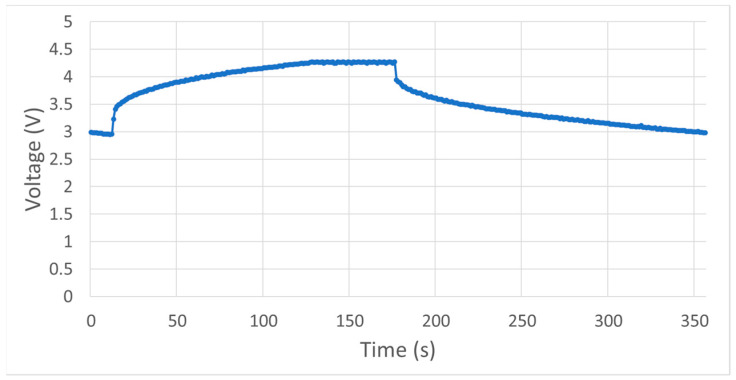
Supercapacitor test.

The supercapacitor was tested by measuring its voltage during the charging and discharging process using Arduino Uno analog input. The charging process was conducted by connecting the system developed with an external power supply, and an Arduino Uno was programmed to monitor the supercapacitor voltage. The time required for this process from a voltage of 3.2–4.2 V was 85 s. Meanwhile, the discharging process involved using the supercapacitor to power the VOCs sensor node and the time required to drive the minimum voltage for the system at 3.2V was 139 s, as indicated in Figure 3. The supercapacitor charging capacity was found to be 2.7 mAh using Equation (3) and the data tested.

Supercapacitor charging capacity (Q_sp_) [32]:

(3) $$Q_{\mathrm{sp}}=\frac{C\left(V_{c}-V_{d}\right)}{3600}$$

where:

Q_sp_ = supercapacitor charging capacity in Amper.hour (Ah).
C = capacitance in Farad (F = A.s/V).
V_c_ = capacitor charged voltage in Volt (V).
V_d_ = capacitor discharged voltage in Volt (V).

#### 2.4.3. Wireless Module

**Figure 4:**
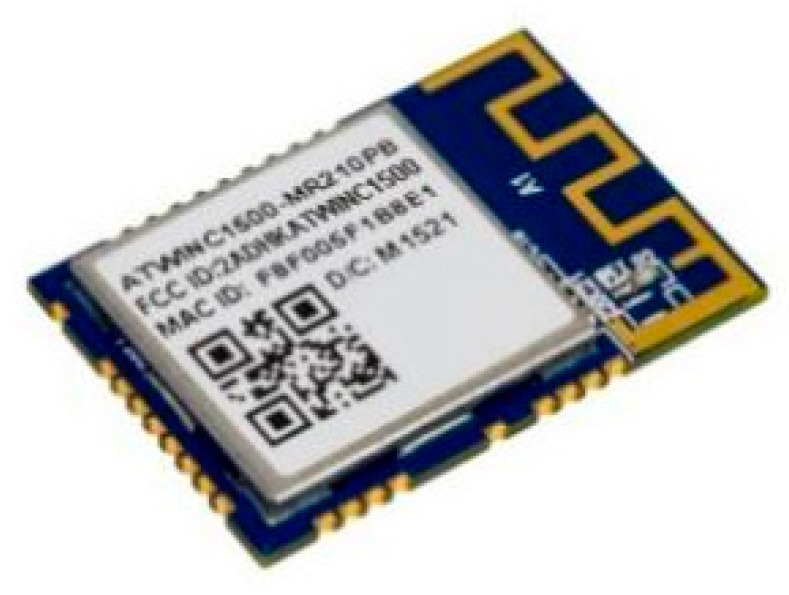
WINC1510 Wi-Fi module.

All the data measured by the VOCs sensor node are designed to be sent to the server through a Wi-Fi connection, which is expected to be maintained as long as the system is working and reinitialized every time the system restarts. The WINC1510 Wi-Fi module used is presented in Figure 4.

This module was selected due to its low power consumption, which is associated with the application of 802.11 b/g/n IoT, which is a standard optimized for low-power IoT applications. It is also equipped with a low-noise amplifier (LNA), power amplifier (PA), switch, and power management. Moreover, the module is small in design and uses a micro co-ax (U.FL) connector for an external antenna and SPI to interface with a microcontroller.

#### 2.4.4. Indoor Air Quality (IAQ) Sensor

**Figure 5:**
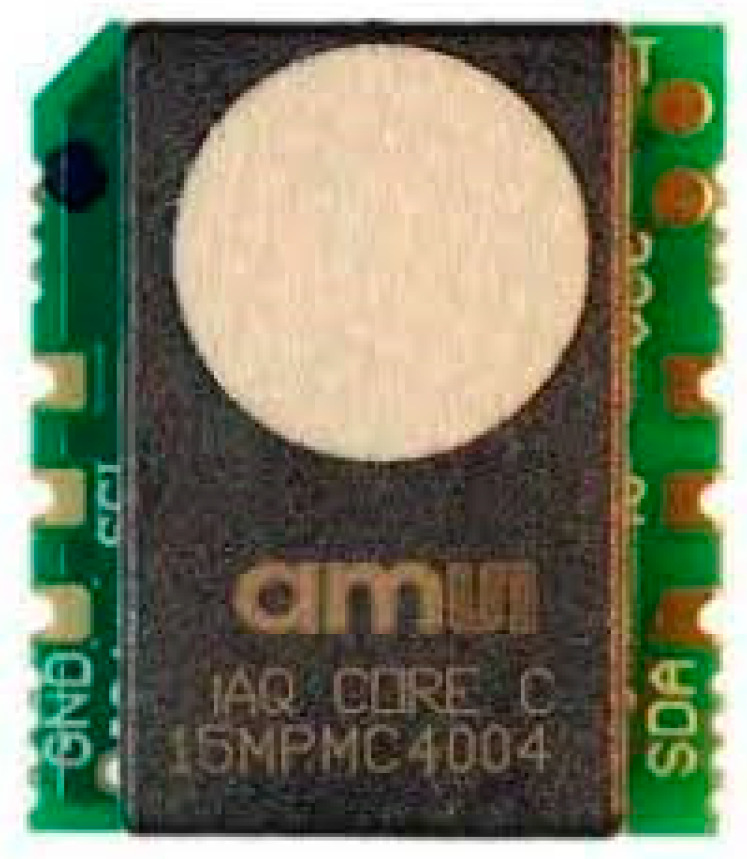
IAQ sensor.

The IAQ sensor module, which has the ability to measure VOCs levels and provide CO_2_ equivalent and TVOCs equivalent predictions, was used in this system. This sensor uses IAQ-Core, which has I_2_C protocol communication, produces calibrated TVOCs and CO_2_ equivalent values [31], and was integrated with the main board of the sensor node. The image of the IAQ sensor is presented in Figure 5.

#### 2.4.5. Software

The final target of the system is to be able to monitor the indoor TVOCs value in real time, and data visualization is necessary to make it easier for users to read the data. Therefore, the Adafruit Dashboard was used to display the data sent by the VOCs sensor node. This dashboard is a platform developed by adafruit.io and observed to be capable of receiving and visualizing data according to the user’s design. The system algorithm applied to monitor the indoor TVOCs value is described as follows (Algorithm 1):
**Algorithm 1** The logical flow of VOCs sensor node sensing process**Start** program while sensor node is powered Initialize Wi-Fi connection. If Wi-Fi connected to internet:  Call function to measure air quality.  Send CO_2_ and TVOCs data.  Display CO_2_ and TVOCs data to IoT dashboard Or Else:  Retry connection to the internet Wi-Fi. Call function to low power mode. Enable alarm and interval start counting. If interval reached:  Call function to system wake-up.   Disable alarm and reset the interval.  Return to measure air quality and repeat. Or Else:  Interval counting. End program while sensor node power is low.

## 3. Results and Discussion

The prototype was verified by testing the component functions, validating the measurement results, calibrating, analyzing the current, voltage, and power consumption, as well as the processing time, and testing the data visualization process. A comprehensive analysis was required to ensure the system developed operates according to the specifications in the design.

### 3.1. Device Implementation

**Figure 6:**
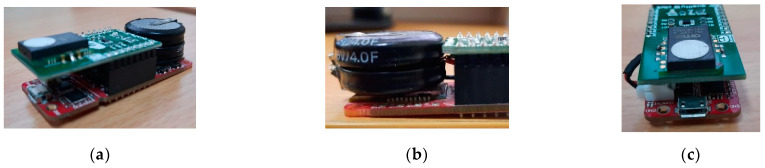
VOCs sensor node prototype: (**a**) side view, (**b**) energy source (supercapacitor), and (**c**) front view.

Figure 6 shows the VOCs sensor node prototype assembled and ready to be used. The compact and minimalist design allows for the sensor nodes to be implemented in small spaces.

### 3.2. Functionality Test

**Figure 7:**
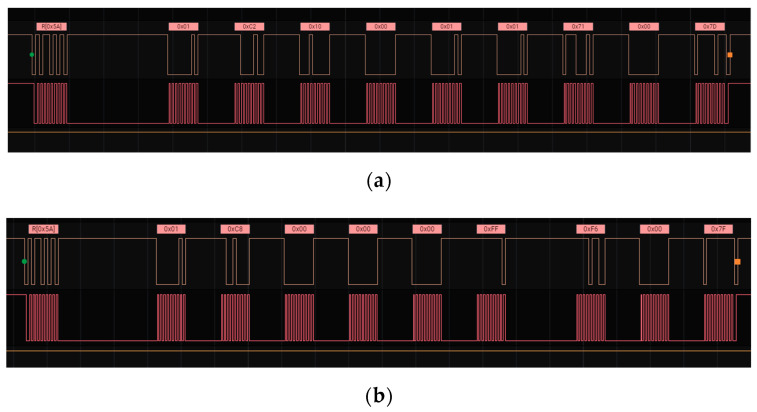
(**a**) Response 0x10 means the sensor is warming up, and (**b**) response 0x00 means the sensor is working well.

Each component used in the VOCs sensor node was tested for functionality to ensure that they can work properly. The tests were conducted on four main blocks including the energy storage, sensor, processor, and wireless, while the power source was evaluated by measuring the charging and discharging process. The functionality of the supercapacitor is based on its ability to keep the power within a voltage rating according to specifications. Moreover, the mainboard was tested simultaneously with the sensor and Wi-Fi, and its functionality was based on its ability to read the sensor. Figure 7 shows the logic analyzer analysis showing that the mainboard can read the data sent by the VOCs sensor through the I_2_C interface.

The Wi-Fi block was assessed by scanning available wireless and connection ability. The results of the functionality test are presented in the following Table 3.

**Figure 8:**
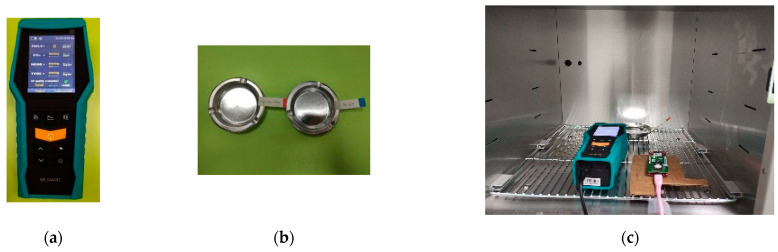
VOCs validation: (**a**) VOCs measurement instrument, (**b**) test object, and (**c**) test configuration.

The VOCs sensor node was validated by comparing the value of its readings with a validator. An industrial portable air quality monitor (BR-Smart BLATN) was used as the validator, as shown in Figure 8a, while the test objects or materials in Figure 8b were used in the test to produce high TVOCs pollutants such as detergent, floor cleaner, and perfume. It is important to note that the VOCs sensor node, industrial portable air quality monitor, and the materials were also placed in a controllable test equipment chamber during the test, as shown in Figure 8c.

The results of the validation process presented in Table 4 showed that the reading values of the VOCs sensor node are close to those of the validator, with the error observed to range from −43 ppb to −20 ppb for the measurement values of 211 ppb to 354 ppb, respectively.

### 3.3. Power Requirement Test

The power was measured to determine the consumption rate of the VOCs sensor node. It is important to note that pre-measurements, such as calibration and validation, are necessary to ensure the accuracy of the tests. Moreover, the power requirement was tested using a power sensor module.

#### 3.3.1. Power Sensor Test

**Figure 9:**
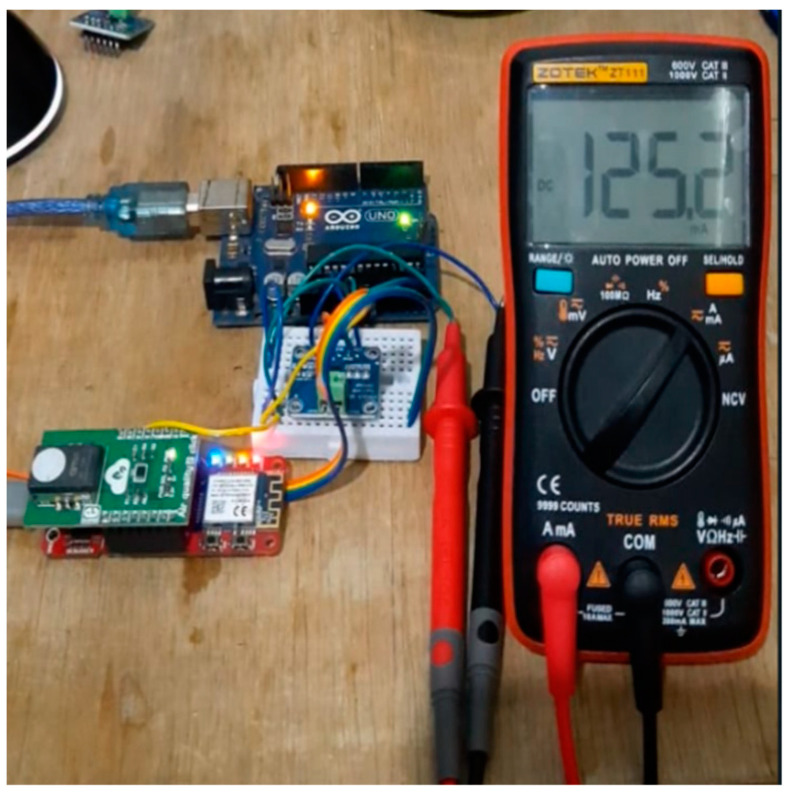
Power measurement.

The 1NA219 sensor was used to measure the current and voltage of the VOCs sensor node as well as the power value. This involved measuring the voltage across 0.1 Ω, 1% sense resistor, and 0.1 mA resolution with maximal current at ±400 mA when the internal gain was set at div8. Moreover, I_2_C was used as the interface to communicate with the processor while Arduino Uno was applied to read the INA219 sensor data output, as shown in Figure 9, to avoid interfering with the measurement of the power on the load.

The results of the 1NA219 sensor were validated as a pre-measurement stage in the process of determining the power in the system. The validation was conducted by comparing the values of the current and voltage measured using the 1NA219 sensor with those obtained from the multimeter. It was discovered that the 1NA219 sensor has a high accuracy lower than 1% for current as shown in Table 5, while the voltage measurement accuracy was 0.33% as indicated in Table 6.

#### 3.3.2. Power Measurement

**Figure 10:**
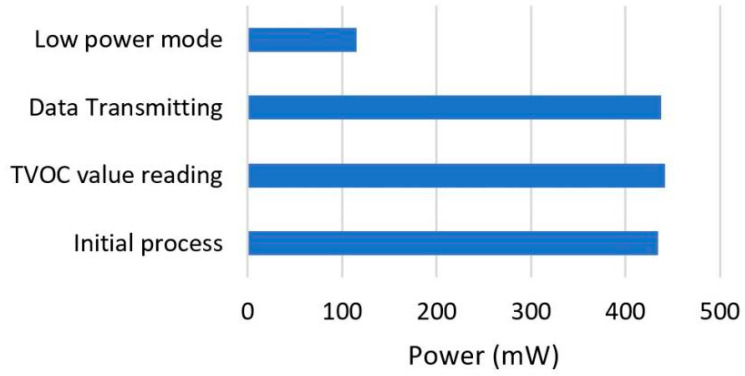
Power measurement data.

The power on the VOCs sensor nodes was measured based on the algorithms described in the Software subsection. The system has four processes in one cycle interval, which include the initial process, reading of TVOCs values, the transmission of data, and low power mode. The initial process is the first step the system takes when it is turned on and all the components including the Mainboard, sensors, and Wi-Fi are initiated. This stage is usually conducted once as long as the system is running and reinitiated when the system restarts. Moreover, the TVOCs value reading stage involves the measurement of the TVOCs pollutants through the IAQM sensor to produce calibrated TVOCs and CO_2_ equivalent values [31] that are sent through the server during the data transmitting stage. The successful completion of the data transmission is normally followed by the activation of the low power mode for 30 min by the system, after which the mode is disabled and the TVOCs value reading stage is repeated for the subsequent data. Figure 10 shows that the highest power consumed was approximately 441.83 mW from the TVOCs value reading stage. The value reduced drastically to 114.16 mW when the low power mode was enabled, and this means the mode is important in autonomous indoor TVOCs monitoring. It is also important to note that the data transmission interval is the key to the lifetime of a system working with a specific energy storage capacity. The system was designed to work on low power mode when no data are being transmitted to reduce the power consumption rate.

#### 3.3.3. Processing Time Consumption

**Figure 11:**
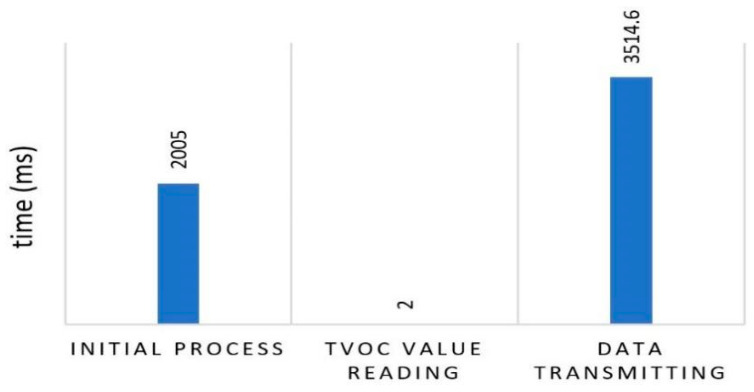
Time measurement.

The processing time was evaluated in each process as observed with the power measurement. The time interval was set at 30 min considering the changes in the relatively fixed values and the fact that there was no need for quick monitoring. Figure 11 shows that the time required for the initial process was approximately 2005 ms with most observed to be for the wireless initialization. The TVOCs value reading process required 2 ms, while the transmission of the two outputs data including CO_2_ and TVOCs values to the server required approximately 3514.6 ms. It is important to note that these are the average values from the five processing rounds conducted.

#### 3.3.4. Power Source Capacity

**Figure 12:**
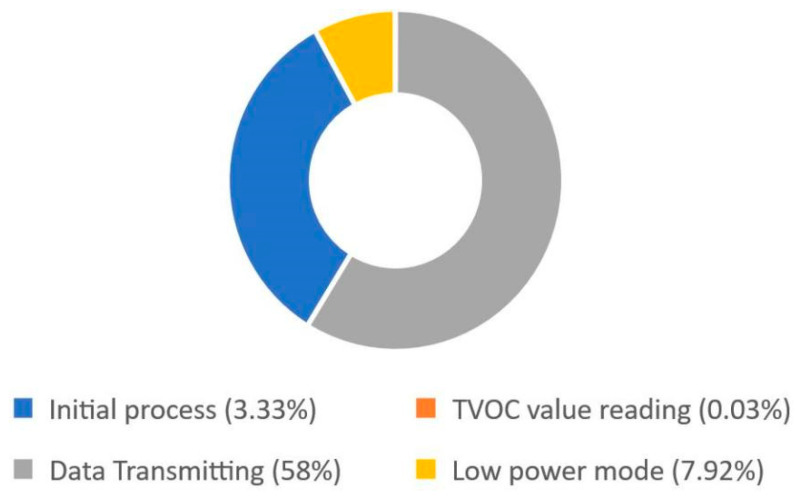
Power source capacity percentage.

The VOCs sensor node reads and transmits the data at the appropriate time interval. Table 7 shows that the highest power was consumed during the data transmitting process with 0.13 mAh, which is 58% of the overall power consumed in one cycle of sensor node operation as indicated in Figure 12. However, the 2.7 mAh capacity of the supercapacitor was reached in just 139 ms, and this was observed to be a good step in conducting further studies on the application of supercapacitors as an alternative power source.

### 3.4. User Interface

**Figure 13:**
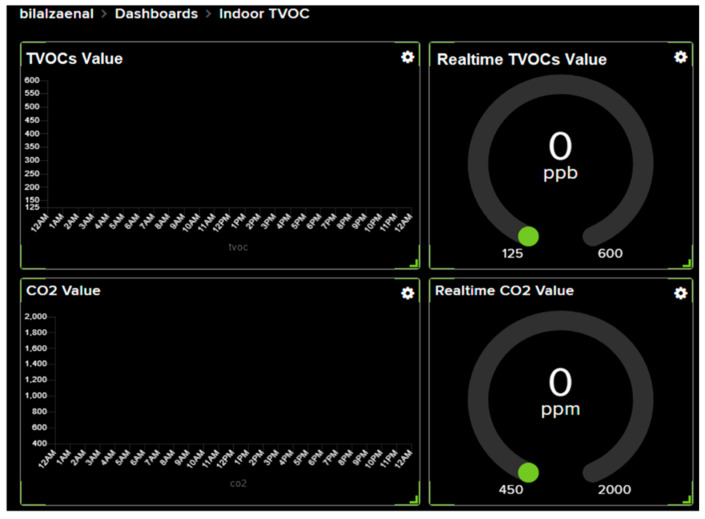
Dashboard design.

Figure 13 shows the dashboard design to visualize indoor TVOCs value with Message Queuing Telemetry Transport (MQTT) used as the communication protocol between the VOCs sensor node and the dashboard to allow the system to be designed to work in real time.

### 3.5. VOCs Field Experiment

#### 3.5.1. Configuration

**Figure 14:**
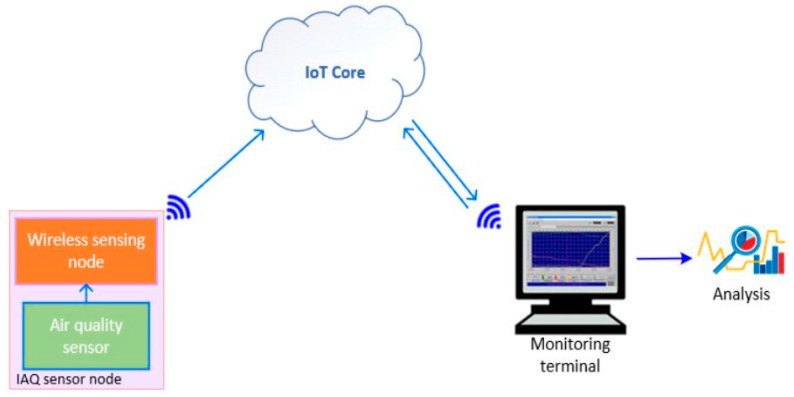
System configuration.

The system configuration consists of a sensor node, a server, and a monitoring terminal as shown in Figure 14.

The sensor node was used to read the TVOCs and CO_2_ values, which were further transmitted to the Adafruit server through an internet connection. It is possible to access the monitoring dashboard from anywhere and at any time through a computer or smartphone on the client side. The TVOCs and CO_2_ parameter values displayed on the dashboard were t used in determining the air quality level.

#### 3.5.2. Result

**Figure 15:**
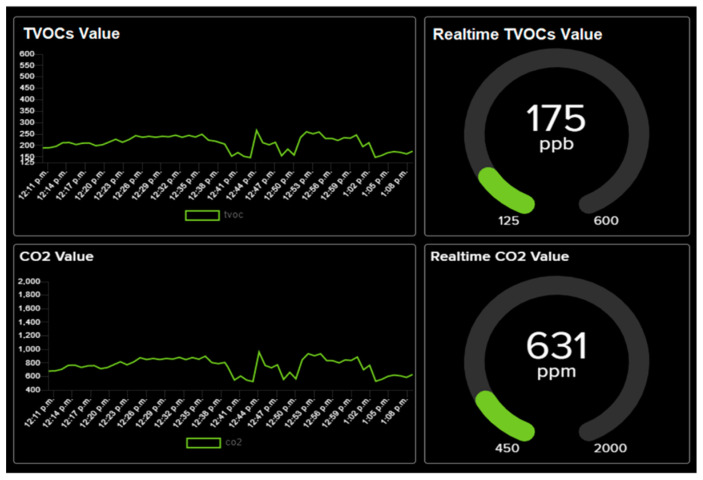
TVOCs and CO_2_ measurement results on the monitoring terminal dashboard.

Figure 15 shows the TVOCs and CO_2_ readings on the dashboard in the university laboratory with the TVOCs real-time value recorded to be 175 ppb, which means it is at a concentration level 2 as shown in Table 1, thereby indicating that the air quality is good.

**Figure 16:**
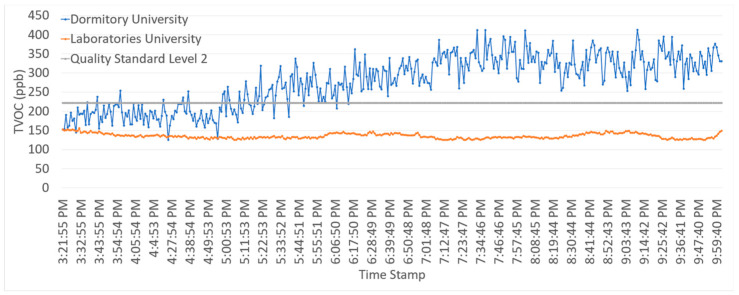
TVOCs measurement results.

The sensor was tested at two different locations as described in Table 8 and observed to be working effectively. Figure 16 shows the IAQ sensor value for 400 min and the laboratory was discovered to have an average TVOCs value below the quality standard of level 2 (<222 ppb), while the dormitory was above the quality standard as defined in Table 1.

## 4. Conclusions

An autonomous wireless VOCs sensor node prototype with a compact size design and low power algorithm was developed in this study. The total power consumption of the node was recorded to be 0.22 mAh for one cycle of sensor node operation, which includes the initial process, TVOCs value reading, data transmission, and low power mode with a time interval of 30 min. The highest power, 0.13 mAh, which is 58% of the total power consumed in the system, was observed to be consumed during the data transmission process. Moreover, the supercapacitor with 10F capacitance used was able to drive the VOCs sensor node for 139 s, and it is possible to extend its lifetime by either increasing the capacity or harvesting micro energy from the indoor environment. The experiment results also showed that TVOCs and CO_2_ values were successfully measured and displayed on the monitoring terminal dashboard through the internet connection.

## Figures and Tables

**Table 1:** TVOCs’ level of concentration [9].

TVOCs Level μg⁄m^3^	TVOCs Level (ppb)	Level of Concentration	Air Quality
<300	<67	Level 1	Excellent
300–1000	67–222	Level 2	Good
1000–3000	222–665	Level 3	Moderate
3000–10,000	665–2218	Level 4	Poor
10,000–25,000	2218–5545	Level 5	Unhealthy

**Table 2:** System specification.

Parameters	Value	Remarks
Power	3.3–5.5 V	Supercapacitor
Interface	I_2_C, Wi-Fi	
Sensing range [31]	450–2000 ppm	CO_2_ equivalents
	125–600 ppb	TVOCs equivalents

**Table 3:** Functionality test results.

Block	Testing Parameter	Result
Power source	Charging and discharging process	Good
Supercapacitor		Good
Processor	Reading the sensor and wireless connection	Good
Wireless	Scanning and connection ability	Good

**Table 4:** Validation results.

Test Objects	TVOCs Value (ppb)		
	VOCs Sensor Node	Validator	Error
Indoor air condition	211	254	−43
Detergent	278	325	−47
Floor Cleaner	305	342	−37
Perfume	354	374	−20

**Table 5:** Current measurements.

Load Test	Current Value (mA)		
	1NA219	Multimeter	Accuracy (%)
VOCs node sensor	119.90	120.02	0.10
VOCs node sensor with activated 4 LED	125.90	125.20	0.56
VOCs node sensor with transmitting data to the cloud	121.50	120.50	0.83

**Table 6:** Voltage measurements.

Load Test	Voltage Value (V)		
	1NA219	Multimeter	Accuracy (%)
VOCs node sensor (VCC)	3.27	3.3	0.33

**Table 7:** Power source capacity.

Process	Power (mA)	Time (ms)	Power Source Capacity (mAh)
Initial process	132.90	2005.00	0.07
TVOCs value reading	135.20	2.00	0.01
Data transmitting	134.00	3514.60	0.13
Low power mode	35.30	1800.00	0.02
Total power consumption			0.22

**Table 8:** The main characteristics of the measurement area.

Measurement Area	Location	Room Description
University	Laboratories of University	Using air conditioner. Far from outside area. Some stuff inside the room: whiteboard, computer, printer, measurement devices, plastic chairs, wood tables, and aluminum cupboards.
University	Dormitory of University	Located nearby the road. Nearby residential and building. Does not use air conditioner. Rarely gets fresh air. Some stuff inside the room: spring bed mattress, pillow, wooden table, wooden chair, wooden cupboard, carpet, and some chemical liquids including perfume, skincare, floor cleaner, and detergent.

## Data Availability

The data presented in this study are available at the request of the corresponding author.
